# The Gene Ontology (GO) Cellular Component Ontology: integration with SAO (Subcellular Anatomy Ontology) and other recent developments

**DOI:** 10.1186/2041-1480-4-20

**Published:** 2013-10-07

**Authors:** Paola Roncaglia, Maryann E Martone, David P Hill, Tanya Z Berardini, Rebecca E Foulger, Fahim T Imam, Harold Drabkin, Christopher J Mungall, Jane Lomax

**Affiliations:** 1 European Molecular Biology Laboratory, European Bioinformatics Institute (EMBL-EBI), Wellcome Trust Genome Campus, Hinxton CB10 1SD, UK; 2 The Gene Ontology Consortium, European Molecular Biology Laboratory, European Bioinformatics Institute (EMBL-EBI), Wellcome Trust Genome Campus, Hinxton CB10 1SD, UK; 3 Department of Neurosciences, Center for Research in Biological Systems, University of California, San Diego, CA, USA; 4 The Jackson Laboratory, Bar Harbor, ME 04609, USA; 5 The Arabidopsis Information Resource, Carnegie Institution for Science, Department of Plant Biology, Stanford, CA 94305, USA; 6 Genomics Division, Lawrence Berkeley National Laboratory, Berkeley, CA, USA; 7 Protein Ontology, Center for Bioinformatics & Computational Biology, Department of Computer and Information Sciences, University of Delaware, 15 Innovation Way, room 205, Newark DE19711, USA

**Keywords:** Gene ontology, Cellular component ontology, Subcellular anatomy ontology, Neuroscience, Annotation, Ontology language, Ontology integration, Neuroscience information framework

## Abstract

**Background:**

The Gene Ontology (GO) (http://www.geneontology.org/) contains a set of terms for describing the activity and actions of gene products across all kingdoms of life. Each of these activities is executed in a location within a cell or in the vicinity of a cell. In order to capture this context, the GO includes a sub-ontology called the Cellular Component (CC) ontology (GO-CCO). The primary use of this ontology is for GO annotation, but it has also been used for phenotype annotation, and for the annotation of images. Another ontology with similar scope to the GO-CCO is the Subcellular Anatomy Ontology (SAO), part of the Neuroscience Information Framework Standard (NIFSTD) suite of ontologies. The SAO also covers cell components, but in the domain of neuroscience.

**Description:**

Recently, the GO-CCO was enriched in content and links to the Biological Process and Molecular Function branches of GO as well as to other ontologies. This was achieved in several ways. We carried out an amalgamation of SAO terms with GO-CCO ones; as a result, nearly 100 new neuroscience-related terms were added to the GO. The GO-CCO also contains relationships to GO Biological Process and Molecular Function terms, as well as connecting to external ontologies such as the Cell Ontology (CL). Terms representing protein complexes in the Protein Ontology (PRO) reference GO-CCO terms for their species-generic counterparts. GO-CCO terms can also be used to search a variety of databases.

**Conclusions:**

In this publication we provide an overview of the GO-CCO, its overall design, and some recent extensions that make use of additional spatial information. One of the most recent developments of the GO-CCO was the merging in of the SAO, resulting in a single unified ontology designed to serve the needs of GO annotators as well as the specific needs of the neuroscience community.

## Background

The Gene Ontology (GO) [1,2] contains a set of terms for describing the activity and actions of gene products across all kingdoms of life. Each of these activities is executed in a cellular location or a location outside in the vicinity of a cell. In order to capture this context, the GO includes, since its inception, a sub-ontology called the Cellular Component Ontology (GO-CCO). GO-CCO terms describe parts of cells and structures associated with cells throughout the taxonomy range. The primary use of this ontology is for GO annotation, but it has also been used for phenotype annotation. Another ontology with a similar scope to the GO-CCO is the Subcellular Anatomy Ontology (SAO) [3], part of the Neuroscience Information Framework Standard (NIFSTD) [4] suite of ontologies. The SAO covers cellular components in the domain of neuroscience and was designed as a model for describing relationships among subcellular structures that would be encountered in an electron micrograph, for example a neuropil. In the nervous system, there are numerous examples of named subcellular structures that are composed of parts of multiple cell types, *e.g.*, synapses, the Node of Ranvier, the *glia limitans*. SAO thus has a richer set of spatial relationships than the GO, modeled in part after the Foundational Model of Anatomy (FMA) [5].

At the time the SAO was constructed, circa 2005–2006, tools for import and reuse of existing ontologies were limited; in addition the SAO was composed in OWL (Web Ontology Language), while the GO-CCO was in OBO (Open Biomedical Ontologies) format. At this time, the semantics of OBO format were not yet aligned with those of OWL. Thus, the SAO had developed an independent set of cell component terms, with a heavy focus on those encountered in the nervous system. More recently, with the advent of a more detailed specification of OBO format (which clarifies the semantics of OBO format as a subset of OWL2) and the development of OBO/OWL converters, the native format of an ontology is less relevant. This has allowed us to work together on the same ontology by incorporating the SAO into the GO-CCO.

The SAO was used primarily within prototype segmentation and annotation tools developed for electron tomography data [6] to enhance search within the NIF across federated data [4] and, as described below, to annotate data derived from imaging and the literature on phenotypes associated with neurodegenerative disease [7]. To ensure that these annotations are not lost, NIF maintains a mapping between SAO and GO-CCO within a bridge file (for details on the use of bridge files in NIFSTD, see [8]).

In this paper, we describe an overview of the GO-CCO, a description of the amalgamation of the GO-CCO with the SAO, followed by a sketch of how the GO-CCO fits in with other ontologies. The last part of the paper describes applications and uses of the GO-CCO. Our aim is to provide a single unified cellular component ontology that can serve the needs of a diverse scientific community. The biomedical and bioinformatics communities may also benefit from the links between the GO-CCO and other ontologies.

The URL for the Gene Ontology (GO) is http://www.geneontology.org/. GO files are publicly available for download at http://geneontology.org/GO.downloads.ontology.shtml.

### Overview of the Cellular Component Ontology

**Figure 1:**
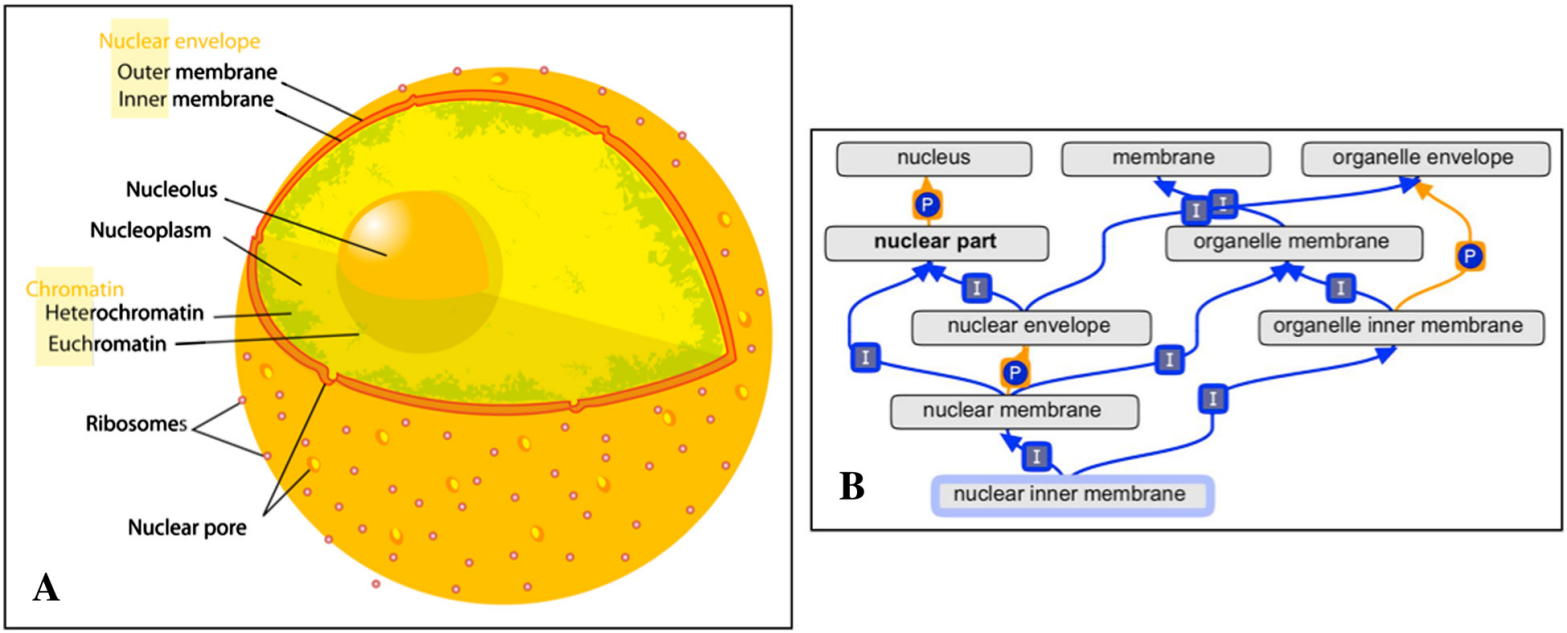
**Diagram and ontology placement of 'nuclear inner membrane'. (A)** Diagram of human cell nucleus, including the nuclear inner membrane. (Taken from Wikimedia commons, http://upload.wikimedia.org/wikipedia/commons/thumb/3/38/Diagram_human_cell_nucleus.svg/2000px-Diagram_human_cell_nucleus.svg.png). **(B)** Placement of the Gene Ontology term GO:0005637 'nuclear inner membrane', drawn using the ontology editing tool OBO-Edit (see ‘Methods’). Due to space limitations, not all ancestor and descendant terms are shown. Is_a links are indicated by "I"; part_of links are indicated by "P" (see main text for explanation).

The Cellular Component Ontology describes subcellular structures and macromolecular complexes. GO-CCO terms may thus be used to annotate cellular locations of gene products. Examples of cellular components include ‘nuclear inner membrane’ (Figure 1) and the ‘ubiquitin ligase complex’, with several subtypes of this complex represented as descendants. The GO-CCO is not taxonomically restricted, and includes terms for both core components found across all domains of life (for example, the species-generic ‘chromosome’) and components specific to particular lineages (for example, ‘Nebenkern’, a mitochondrial formation found in insects, and ‘thylakoid’, a compartment inside chloroplasts and cyanobacteria).

The two core relationship types used in the GO-CCO are ‘is_a’ and ‘part_of’. The ‘is_a’ relation (also known as “SubClassOf”) represents the relationship between a more generic term and a specialized term (for example, between ‘membrane’ and ‘plasma membrane’), whereas the ‘part_of’ relationship describes how sub-structures are assembled into larger structures (for example, between ‘nucleolus’ and ‘nucleus’) [9].

Generally, experimental results or computational predictions support statements that a gene product is located in or is a subcomponent of a particular cellular component. The GO-CCO includes multi-subunit enzymes and other protein complexes, but not individual proteins or nucleic acids. (Terms describing protein complexes are further discussed below.) Whilst the GO-CCO includes cell structures, it excludes cell types, which are instead represented in the Cell Ontology (CL) [10] or the plant cell branch of the Plant Ontology (PO) [11]. The GO-CCO also excludes multicellular anatomical terms, with such structures being described by either species-specific ontologies (e.g., Zebrafish anatomy ontology [12], Mouse gross anatomy ontology [13]) or taxonomically broad anatomical ontologies (e.g., Uberon [14], PO).

The 2013-06-18 release of the GO contains 3332 CC ontology terms. Approximately half of these terms represent protein complexes, with the other half representing larger units.

### Amalgamation with SAO

The SAO was incorporated into the Neuroscience Information Framework standard ontologies when they were originally assembled (NIFSTD) [15]. The NIF project [16] was charged with providing a semantic framework for describing and searching neuroscience data. NIFSTD was built from community ontologies when possible, but as noted above, working with community ontologies was often a challenge when the project began. Over the course of the project, NIF gradually replaced its custom ontologies with more general community ontologies when they became available, both to benefit from the continued enrichment of these ontologies by the life sciences community and to ensure that annotations in the NIF would be compatible with the larger life sciences community. In this case, a reconciliation of the NIF and the GO-CCO was required. Through this reconciliation, not only would NIF’s data federation and search benefit from the on-going development and extensive use of the GO for annotations, but the community ontologies would become enriched with the neuroscience-specific content developed by NIF. The SAO-GO-CCO integration is an example of this type of harmonization.

We started from a list of about 400 terms from the NIF Subcellular Anatomy Ontology (SAO) representing sub-cellular locations that required integration into the GO-CCO. GO editors carefully examined the list and considered each term as appropriate. The following categories were identified:

1) Terms that were already in the GO;

2) Terms that needed to be added to the GO;

3) Terms that were out of scope for the GO.

Terms that were already in the GO were:

- high-level GO-CCO terms that were included in the SAO to provide some structure (*e.g.* ‘plasma membrane’);

- recent additions to the GO that had not yet been documented in SAO; in these cases, the NIFSTD IDs have been included in the GO as database cross-references;

- present in the GO under a different primary name than used by the SAO/NIF; where appropriate, the missing SAO names have been added to the GO as synonyms, along with their NIFSTD IDs.

**Figure 2:**
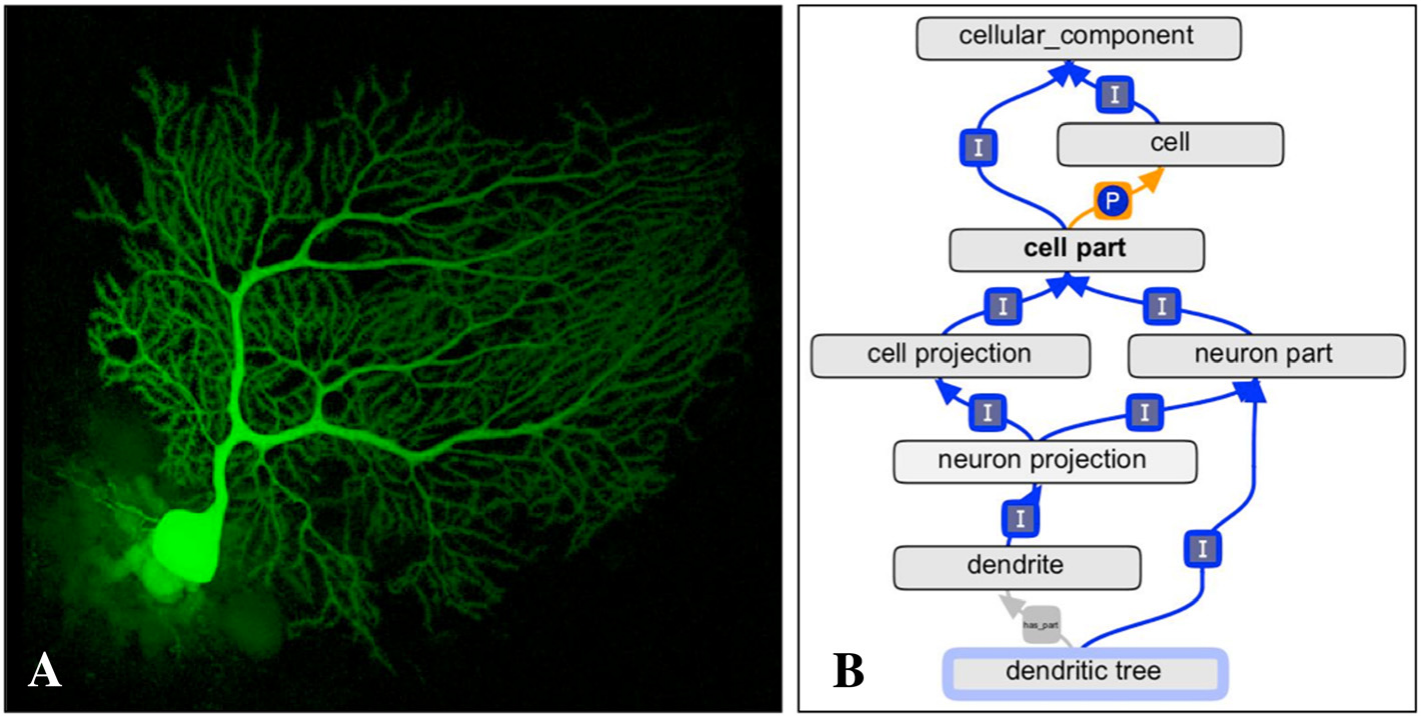
**Image and ontology placement of ‘dendritic tree’. (A)** Dendritic tree from a Purkinje neuron from mouse cerebellum injected with Lucifer Yellow and imaged using confocal microscopy. (Taken from the Cell Centered Database (CCDB), http://ccdb.ucsd.edu/sand/main?mpid=3&event=displayRecon). **(B)** Placement of the newly added Gene Ontology term GO:0097447 'dendritic tree', drawn using the ontology editing tool OBO-Edit (see ‘Methods’). Due to space limitations, not all ancestor and descendant terms are shown. Is_a links are indicated by "I"; part_of links are indicated by "P" (see main text for explanation). Has_part links are also discussed in the main text.

SAO terms that needed to be added to the GO were created, and their NIFSTD IDs recorded as database cross-references. Definitions for the SAO terms were taken from the SAO where available, edited if necessary, or written by GO editors based on the literature or after consultation with SAO editors. 97 new terms were added to the GO (*e.g.* ‘dendritic tree’ (Figure 2), ‘ribbon synapse’); their full list is shown in Table 1.

**Table 1:** Terms added to GO-CCO from SAO

GO:0043220	Schmidt-Lanterman incisure
GO:0044224	juxtaparanode region of axon
GO:0044225	apical pole of neuron
GO:0044226	basal pole of neuron
GO:0044280	subplasmalemmal coating
GO:0044284	mitochondrial crista junction
GO:0044285	bridge contact site
GO:0044286	peg and socket contact
GO:0044288	puncta adhaerentia
GO:0044289	contact site
GO:0044290	mitochondrial intracristal space
GO:0044291	cell-cell contact zone
GO:0044292	dendrite terminus
GO:0044293	dendriole
GO:0044294	dendritic growth cone
GO:0044295	axonal growth cone
GO:0044296	dendritic tuft
GO:0044299	C-fiber
GO:0044300	cerebellar mossy fiber
GO:0044301	climbing fiber
GO:0044302	dentate gyrus mossy fiber
GO:0044303	axon collateral
GO:0044304	main axon
GO:0044305	calyx of Held
GO:0044307	dendritic branch
GO:0044308	axonal spine
GO:0044309	neuron spine
GO:0044352	pinosome
GO:0044754	autolysosome
GO:0097407	Bunina body
GO:0097408	fibrillary inclusion
GO:0097409	glial cytoplasmic inclusion
GO:0097412	hyaline inclusion
GO:0097413	Lewy body
GO:0097414	classical Lewy body
GO:0097415	cortical Lewy body
GO:0097416	Lewy body-like hyaline inclusion
GO:0097417	nematosome
GO:0097418	neurofibrillary tangle
GO:0097419	Pick body
GO:0097420	skein-like inclusion
GO:0097422	tubular endosome
GO:0097423	mitochondrion-associated adherens complex
GO:0097424	nucleolus-associated heterochromatin
GO:0097425	smooth endoplasmic reticulum part
GO:0097426	glial filament
GO:0097427	microtubule bundle
GO:0097433	dense body
GO:0097440	apical dendrite
GO:0097441	basilar dendrite
GO:0097442	CA3 pyramidal cell dendrite
GO:0097443	sorting endosome
GO:0097444	spine apparatus
GO:0097445	presynaptic active zone dense projection
GO:0097447	dendritic tree
GO:0097448	spine mat
GO:0097449	astrocyte projection
GO:0097450	astrocyte end-foot
GO:0097451	glial limiting end-foot
GO:0097453	mesaxon
GO:0097454	Schwann cell microvillus
GO:0097455	spiny bracelet of Nageotte
GO:0097456	terminal loop
GO:0097457	hippocampal mossy fiber
GO:0097458	neuron part
GO:0097462	Lewy neurite
GO:0097463	gemmule
GO:0097464	thorny excrescence
GO:0097465	somatic spine
GO:0097470	ribbon synapse
GO:0097471	mossy fiber rosette
GO:1901588	dendritic microtubule
GO:1901589	axon microtubule bundle
GO:1990005	granular vesicle
GO:1990006	amorphous vesicle
GO:1990007	membrane stack
GO:1990008	neurosecretory vesicle
GO:1990011	laminated body
GO:1990012	complex laminated body
GO:1990013	presynaptic grid
GO:1990014	orthogonal array
GO:1990015	ensheathing process
GO:1990016	neck portion of tanycyte
GO:1990017	somatic portion of tanycyte
GO:1990018	tail portion of tanycyte
GO:1990024	C bouton
GO:1990025	F bouton
GO:1990026	hippocampal mossy fiber expansion
GO:1990027	S bouton
GO:1990030	pericellular basket
GO:1990031	pinceau fiber
GO:1990032	parallel fiber
GO:1990033	dendritic branch point
GO:1990037	Lewy body core
GO:1990038	Lewy body corona
GO:1990039	hypolemmal cisterna
GO:1990040	sub-surface cisterna

**Figure 3:**
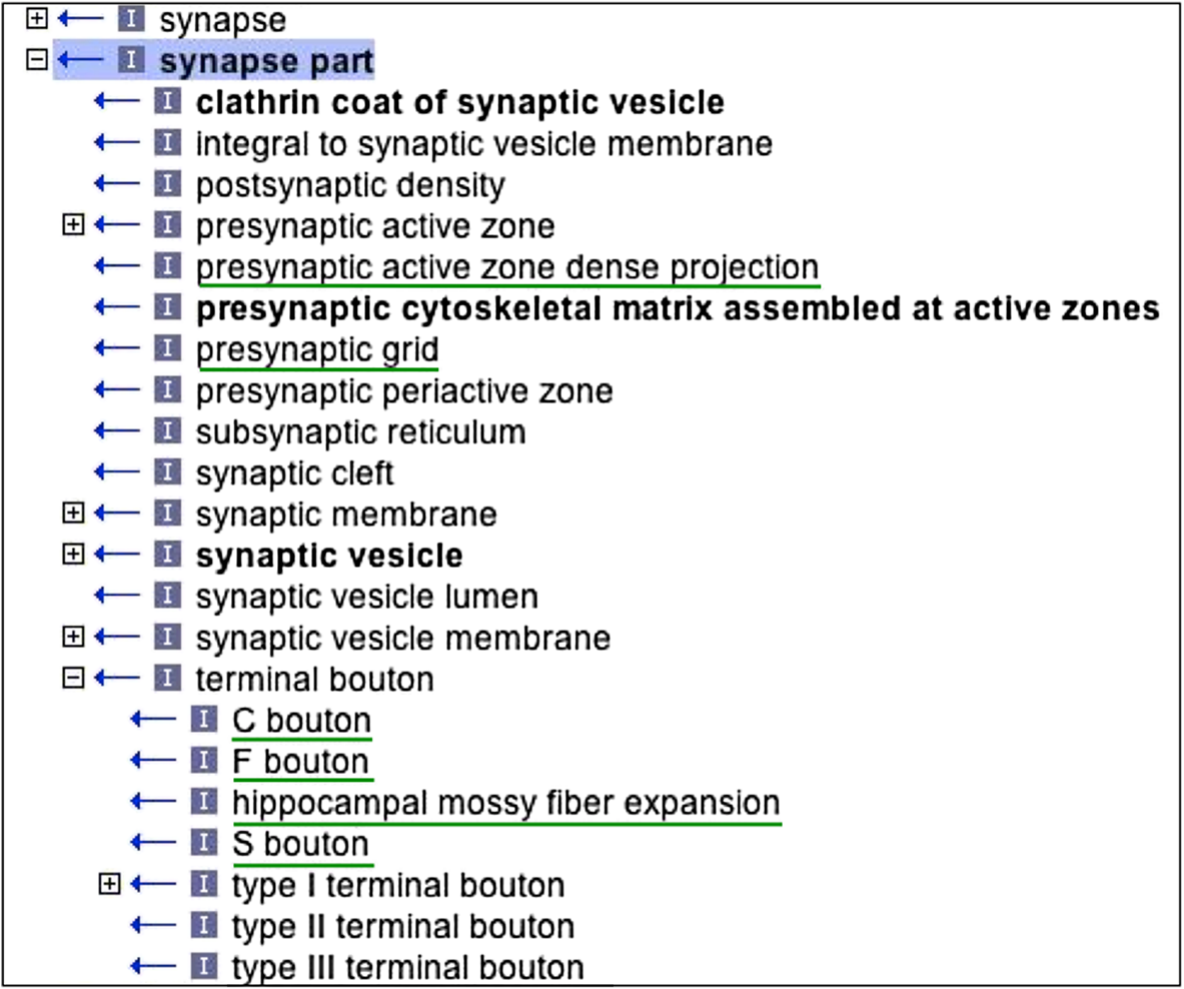
**Representation of 'synapse part' in the Gene Ontology.** Children of the GO term. GO:0044456 'synapse part' are shown using the ontology editing tool OBO-Edit (see ‘Methods’). Terms underlined in green were added as part of the SAO amalgamation. Due to space limitations, not all ancestor and descendant terms are shown. Is_a links are indicated by "I" (see main text for explanation).

The newly added GO-CCO terms (integrated from SAO) include cytoplasmic inclusions such as ‘Lewy body’ and subtypes, cell-type specific variants of structures such as ‘CA3 hippocampus pyramidal cell dendrite’ and terminal boutons such as ‘C bouton’ (Figure 3). Very specific terms such as ‘CA3 hippocampus pyramidal cell dendrite’ have been included because they represent instances with peculiarities that influence their biological role, *e.g.* because they convey particular electrical properties. The full list of newly added terms is available in Table 1.

Some SAO terms represented multicellular anatomical terms, and were therefore out of scope for the GO. Instead, they were suggested as additions to ontologies such as Uberon (*e.g.* ‘axon bundle’, defined as ‘Group of myelinated or unmyelinated axons that group together.’).

### Some recent extensions that make use of additional spatial information

#### Has_part

In addition to is_a and part_of within the GO-CCO, the GO also makes use of additional relationship types, both within the GO-CCO and connecting the GO-CCO to other ontologies.

Recently the *has_part* (BFO:0000051) relation was added to the GO-CCO [17] to represent the composition of components with respect to their sub-components. These relationships add value beyond the reciprocal part_of relationships because they add a dependency of a structure to always have a certain sub-part. While it is true to say that every nucleus is part of a cell at every point during the existence of that nucleus, it is not true that every cell has a nucleus (exceptions including bacterial cells and mammalian erythrocytes) – thus the GO-CCO includes a part_of link between nucleus and cell, but no reciprocal has_part link. Similarly, there is a has_part link between ‘trans splicesomal complex’ and ‘U2 snRNP’, but no reciprocal part_of link. U2 snRNPs are not always a part of a trans splicesomal complex, but every trans splicesomal complex has a U2 snPNP part.

#### Connecting cell components to cell types

To record the connections between terms in the GO-CCO and cell types in the cell type ontology, the GO maintains an additional supplementary bridging ontology called *x-cell-parts.owl*[18] that contains statements such as ‘astrocyte projection’ part_of some ‘astrocyte’. The former term belongs to the GO and the latter to the Cell Ontology (CL) [10,19]. In addition, the Cell Ontology includes links from cell types such as ‘nucleate erythrocyte’ to GO-CCO terms such as ‘nucleus’. These can be found in the full version of the Cell Ontology [20].

#### Connecting biological processes and molecular functions to cell components

Logical definitions (equivalence axioms) are being added to the GO that link the Biological Process (BP) and Molecular Function (MF) branches of the ontology to the GO-CCO [21]. These additional axioms are available in an extended version of the GO called go-plus [22], and allow for fuller reasoning over GO as well as for automation of new term creation using the GO TermGenie system [manuscript in preparation]. This work is ongoing, and axioms are being added using relationships such as *results_in_assembly_of*, *results_in_disassembly_of*, *occurs_in*, *has_start_location*, *capable_of*. Some examples of such relationships in OWL are shown below. For illustrative purposes we use a variant of OWL Manchester Syntax where we include labels in with the identifiers to enhance readability.

Class: ‘GO:0000045 ! autophagic vacuole assembly’ EquivalentTo:‘GO:0022607 ! cellular component assembly’ and results_in_assembly_of some ‘GO:0005776 ! autophagic vacuole’

Class: ‘GO:0000390 ! spliceosomal complex disassembly’ EquivalentTo: ‘GO:0022411 ! cellular component disassembly’ and results_in_disassembly_of some ‘GO:0005681 ! spliceosomal complex’

Class: ‘GO:0006264 ! mitochondrial DNA replication’ EquivalentTo: ‘GO:0006260 ! DNA replication’ and ‘BFO:0000062 ! occurs_in’ some ‘GO:0005739 ! mitochondrion’

Class: ‘GO:0006888 ! ER to Golgi vesicle-mediated transport’ EquivalentTo: ‘GO:0016192 ! vesicle-mediated transport’ and has_target_end_location some ‘GO:0005794 ! Golgi apparatus’ and has_target_start_location some ‘GO:0005783 ! endoplasmic reticulum’

### Terms that describe protein complexes and integration with the Protein Ontology

Of the current 3332 terms in the GO-CCO, 1622 terms are descendants of GO:0032991 macromolecular complex; most of these (1510) are descendants of GO:0043234 protein complex.

Like the rest of GO, protein complexes in the GO-CCO should be applicable to more than one species. This can be difficult to achieve when a complex has been characterized in a single species, or homology among species is unclear. In these cases our aim is to make the term as generic as possible. A protein complex from a particular species is often described in the GO-CCO textual definition, but the scope of that term is not limited to that species. Other resources can use these generic protein-complex terms to classify more specific entities. For example, the Protein Ontology (PRO) [23] makes species-specific protein complex subclasses of the generic GO protein complexes.

**Figure 4:**
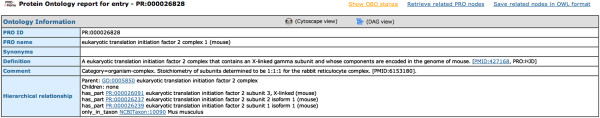
**Protein ontology report for entry PR:000026828.** Ontology information for the Protein Ontology term 'eukaryotic translation initiation factor 2 complex 1 (mouse)', showing parent GO term and *has_part* links to other PR terms [25].

An example of a protein complex, the mouse-specific form of eukaryotic translation initiation factor 2 complex 1, is represented in PRO using the ID PR:000026828 (Figure 4). Each species-specific translation initiation factor complex is composed of specific protein entities, which can be any combination of isoforms, processed forms, or post-translationally modified forms. In this example, the mouse complex is shown. The human complex would be defined using human proteins and would be restricted to the human taxon. Both human and mouse complexes are defined with an *is_a* relationship to the generic GO complex. At present, there are almost 500 protein complex terms in PRO [24].

GO-CCO protein complexes are defined by some combination of their biological function, their subunit composition in one or more species, and their location within the cell. Protein complexes in the GO range from simple dimeric complexes, for example ‘TAP complex’, to complexes having many subunits, for example ‘proteasome complex’. Homodimeric complexes are also included.

In the protein complex branch of the GO-CCO, most protein complexes (729 of 1502) are direct subclasses of ‘protein complex’ itself. This ‘flat’ arrangement is not ideal for the purposes of navigation and data summarization. GO curators are working with the IntAct group [26] to improve the sub-categorization of protein complexes. In cases where a protein complex always contributes to a larger macromolecular structure in a cell we provide a part_of relationship between the protein complex and the larger component. For example, ‘histone deacetylase complex’ is part of the nucleoplasm in the GO. In cases where complexes are found in multiple locations, or move between cell components, the generic protein complex is merely part of ‘cell’.

### Applications of the CC ontology

#### GO Annotation

The primary use of the GO-CCO is to annotate localization of gene products. There are currently 886238 annotations (both experimental and electronic) of gene products from a variety of species. These annotations can be interrogated with GO browsers such as AmiGO 2 [27,28] or QuickGO [29,30]. Annotation describes the process of assigning GO terms to gene products. Annotation can be carried out either automatically or manually. Automated methods provide a fast and efficient way of creating a large set of annotations. For automatic annotation, curators have constructed various mapping files between external features and GO terms. GO annotations are automatically applied to gene products via the mapping files. For example, InterPro entries are manually annotated with terms from the GO [31]. InterPro entry IPR019038 (DNA polymerase subunit Cdc27) contains a mapping to GO:0005634 ‘nucleus’, and any protein that is a member of this InterPro family will receive the annotation GO:0005634. Similarly, UniProtKB entries are manually and electronically tagged with keywords [32] including a cellular component category. A mapping file between UniProtKB keywords and GO terms allows transitive electronic GO annotation of the UniProtKB entry.

Manual annotations are created by curators assessing experimental evidence from published literature. Manual annotations generally result in the use of more specific GO terms. Curators use the experiments or analyses described in a paper to infer the localization of a gene product in a particular cellular compartment. For example, a curator has annotated the human MPV17 mitochondrial membrane protein-like protein (MPV17L; UniProtKB:Q2QL34) with the GO term GO:0005777 'peroxisome' using evidence from Iida et al., [33] who demonstrate co-localization of MPV17L with a fluorescent peroxisomal marker.

Sequence-based analysis can also form the basis for an annotation; GO-CCO annotations can be made based on the presence of a key sequence or structural feature in the gene product. Manual annotations can also be transferred to similar gene products either computationally or when orthology is indicated in the literature. Whether automatic or manual, every annotation is attributed to a source (either a literature reference, a computational analysis or another database) and an evidence code is used to indicate the type of evidence that supports the annotation [34].

**Figure 5:**
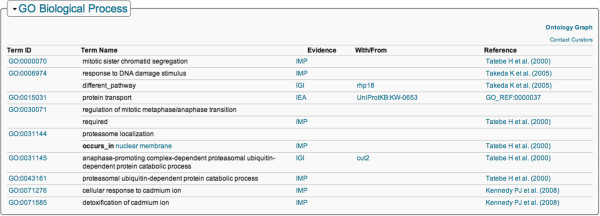
**Refining GO annotations using Gene Ontology CC terms.** The PomBase annotation for the ‘cut8’ gene to ‘proteasome localization’ (a GO biological process term) is further refined by specifying that this localization takes place in the ‘nuclear membrane’ (a GO-CCO term). (Taken from http://www.pombase.org/spombe/result/SPAC17C9.13c#go).

Recently, the GO annotation model has been extended to include contextual annotations. A GO annotation can be further refined using ontology terms from within the GO or other ontologies. For example, the PomBase annotation for the ‘cut8’ gene to ‘proteasome localization’ (a GO biological process term) is further refined by specifying that this localization takes place in the ‘nuclear membrane’ (a GO-CCO term) (Figure 5).

#### Neuronal connectivity

Neuron connectivity maps provide a way to help understand how the nervous system works. The FlyBase anatomy ontology contains a variety of connectivity relationships between neurons in the *Drosophila* nervous system [35] and these relationships are used to deliver powerful queries using the Virtual Fly Brain database [36]. These relationships use the GO-CCO to precisely specify how the cells are connected – for example, the has-pre-synaptic-terminal-in relation is specified using the GO-CCO class ‘post-synaptic membrane’.

#### Image annotation

The Cell Image Library (CIL) is a database of cell images that is indexed using multiple ontologies [6]. The GO-CCO is the ontology that is used to annotate any subcellular structures highlighted in the image. The CIL can be searched using GO terms, and the hierarchy of the GO is incorporated into the query. For example, searching for ‘cytoskeleton’ (GO:0005856) returns images annotated directly with ‘cytoskeleton’, as well as cytoskeletal parts, such as ‘microtubule basal body’ e.g. http://www.cellimagelibrary.org/images/38899.

#### Pathway and interaction databases

The GO-CCO has been used in a variety of pathway databases such as IntAct and Reactome to specify the site in which binding or a molecular event takes place.

IntAct [26], a member of the International Molecular Exchange Consortium (IMEX), uses the GO-CCO to capture molecular interaction data. The IntAct database [37] can record the site of interaction by cross-referencing interaction entries to GO-CCO terms. Over 8000 experimentally-defined IntAct interactions have manually-assigned GO-CCO terms and users can search on interaction cross-references to retrieve, for example, all interactions that occur at the plasma membrane (GO:0005886) or nucleus (GO:0005634).

Because protein function and location are often tightly linked, the manually curated and peer-reviewed pathway database Reactome [38] cross-references GO-CCO terms [39]. Reactome uses a subset of the GO-CCO to annotate the subcellular locations of entities; entities include proteins, nucleic acids, small molecules and subatomic particles, and can be a single molecule or a collection of components such as a macromolecular complex. A GO-CCO term is also a required attribute in the curation of a Reactome event, a biochemical reaction with a defined input (reactant) and output (product), such as the association of two proteins to form a complex, or a catalytic reaction.

#### Annotation of models

The EBI BioModels database [40,41] contains a number of systems biology models represented in SBML (Systems Biology Markup Language) format. SBML provides slots for indicating the compartment of a reaction, resulting in the ability to search for models involving particular cellular components, such as the ‘giant axon’.

#### Enhanced search of multiple resources

The GO-CCO can also be used to search a variety of databases via the Neuinfo interface [16]. For example, searching for data associated with “axoneme” (GO:0005930) [42] returns several data types, including images of axonemes and aging phenotypes associated with axonemes.

#### Phenotypes and disease

Although the GO-CCO, like the rest of the GO, focuses on structures that are found mostly in ‘non-pathological’ contexts, the GO-CCO has applications in bioinformatics analyses of phenotypes and diseases. The Mammalian Phenotype (MP) ontology [43] contains terms for describing abnormalities observed in clinical or model organism experimental settings. Many MP terms such as ‘abnormal mitochondrion morphology’ have been translated to OWL expressions that reference GO-CCO terms such as ‘mitochondrion’, allowing for cross-database phenotype comparisons [44]. The Neurodegenerative Disease Phenotype Ontology (NDPO) [7], with its associated Phenotype Knowledge Base (PKB), uses a model that incorporates descriptions for both human disease phenotypes and those of animal models. Entities are drawn from community ontologies (including the GO-CCO) made available through the Neuroscience Information Framework (NIF) and qualities are drawn from the Phenotype and Trait Ontology (PATO). The resulting phenotype statements describe structural alterations at the subcellular, cellular and gross anatomical levels.

Many diseases are the result of an abnormality within a specific cell component. For example, the disease class ‘ciliopathy’ encompasses a range of disorders such as Bardet-Biedl syndrome and Alström syndrome characterized or caused by an abnormality of the cilium or its subcellular structures. Candidate genes for disorders such as these can be found by scanning GO annotations for gene products that are localized to, or play some role in, the assembly of the relevant cellular components.

Currently the GO-CCO has relationships to taxa of the form ‘only in taxon’ or ‘never in taxon’ [45,46]. For example, the CC ‘plastid’ has a never_in_taxon link to ‘Metazoa’ and ‘Fungi’ supported by a particular publication (in this case [47]). We intend to increase the coverage of GO for certain kinds of unicellular organisms that are currently under-represented in GO. For example, trophozoites such as *Giardia* have characteristic structures such as a 'ventral disc', which can be further subdivided into other parts such as microribbons and microtubular components. These additions will be accompanied by the relevant taxon constraints [45]. Our priority is to include new terms as required for annotation. Other possible areas for extension include dinoflagellates and algae.

## Conclusions

For researchers to be best able to make use of the data available to them, a single system for classification is essential. Prior to this work, the NIF’s SAO and the GO-CCO provided alternative systems for classifying the same data, potentially hindering cross-database analyses. The SAO has now been incorporated into the GO-CCO, which was expanded where necessary, with the result being a single system of classification for subcellular entities across both resources. The SAO was never intended, however, to replicate the GO; rather, it was a means of specifying the relationships among structures encountered in microscopic images. As such, it was a model for describing instances [3,48], rather than intending to serve as a reference ontology. With the improvement in tools for working with community ontologies, in particular, tools to convert between OBO format and OWL (http://oboformat.org) it no longer made sense to maintain the two separately. Rather, the SAO will be rebuilt as an annotation model that imports the GO-CCO for cellular components.

The enhancements to the Cellular Component section of the GO described in this paper will benefit researchers in basic biology, biomedicine and systems biology who use ontologies in their research. The amalgamation of the SAO into the GO-CCO resulted in a single unified ontology designed to serve the broad needs of GO annotators as well as the specific needs of the neuroscience community.

## Methods

### Ontology Development

The SAO was originally developed using Protégé 3, and the GO-CCO developed as part of GO using OBO-Edit [49]. More recently the GO editors have been using a hybrid approach, developing GO using a combination of OBO-Edit, Protégé 4 and TermGenie [manuscript in preparation]. In particular, a supplementary constraints ontology called x-disjoints.owl is maintained in OWL and used as part of the GO continuous integration system [50] using OWL reasoners such as Elk [51]. This ontology contains OWL axioms stating, for example, that a nucleus and a cytoplasm can share no parts.

### Amalgamation

In order to amalgamate the SAO into the GO-CCO, we supplemented the manually maintained mappings between the SAO and the GO-CCO (which had become stale since the SAO was first developed) with automatically-generated mappings based on lexical matching. These were all individually examined by GO editors to determine their accuracy. If considered correct, they were added into the GO-CCO with a database cross-reference to SAO.

For the remaining SAO terms for which no mapping to the GO-CCO could be determined manually or automatically, the GO editors evaluated each to determine if it was appropriate to add to the GO. See the section ‘Amalgamation with SAO’ for more details.

## Availability

The URL for the Gene Ontology (GO) is http://www.geneontology.org/.

Gene Ontology files are publicly available for download [52]. Detailed documentation on the Gene Ontology can also be found online [53]. Researchers wishing to annotate their experiments using GO terms may refer to the GO’s annotation guidelines [54], and contact the GO helpdesk [55].

## Abbreviations

BP: Biological process; CC: Cellular component; CCDB: Cell centered database; GO-CCO: Gene ontology cellular component ontology; CIL: Cell image Library; CL: Cell ontology; GO: Gene ontology; MF: Molecular function; MP: Mammalian phenotype; NDPO: Neurodegenerative disease phenotype Ontology; NIFSTD: Neuroscience information framework standard; OBO: Open biological ontologies; OWL: Web ontology language; SAO: Subcellular anatomy ontology.

## Competing interests

The authors declare that no competing interest exists.

## Authors’ contributions

PR worked on the SAO-GO-CCO amalgamation and drafted the manuscript. DPH and JL contributed to the SAO-GO-CCO amalgamation. MEM and FTI worked on the NIF project, on the SAO and on the CCDB. HD is also a curator for the Protein Ontology and contributed to the section on protein complexes. PR, DPH, TZB, REF, HD, CJM and JL worked on expanding the GO-CCO and linking it to other branches of GO and to external ontologies. MEM, DPH, TZB, REF, HD, CJM and JL helped draft the manuscript. All authors read and approved the final manuscript.
